# Functional Expression and Characterization of Tetrachloroethene Dehalogenase From *Geobacter* sp.

**DOI:** 10.3389/fmicb.2018.01774

**Published:** 2018-08-10

**Authors:** Ryuki Nakamura, Tomohiro Obata, Ryota Nojima, Yohey Hashimoto, Keiichi Noguchi, Takahiro Ogawa, Masafumi Yohda

**Affiliations:** ^1^Department of Biotechnology and Life Science, Tokyo University of Agriculture and Technology, Tokyo, Japan; ^2^Department of Bioapplications and Systems Engineering, Tokyo University of Agriculture and Technology, Tokyo, Japan; ^3^Instrumentation Analysis Center, Tokyo University of Agriculture and Technology, Tokyo, Japan; ^4^Institute of Global Innovation Research, Tokyo University of Agriculture and Technology, Tokyo, Japan

**Keywords:** reductive dehalogenase, tetrachloroethene, *Geobacter*, reconstitution, cobalamin

## Abstract

Reductive dehalogenase (RDase) consists of two parts, RdhA and RdhB. RdhA is the catalytic subunit, harboring a cobalamin cofactor and two Fe–S clusters. RdhA is anchored to the cytoplasmic membrane via the membrane anchoring subunit, RdhB. There are many genes encoding RDases in the genome of organohalide-respiring bacteria, including *Dehalococcoides* spp. However, most genes have not been functionally characterized. Biochemical studies on RDases have been hampered by difficulties encountered in their expression and purification. In this study, we have expressed, purified and characterized RdhA of RDase for tetrachloroethene (PceA) from *Geobacter* sp. PceA was expressed as a fusion protein with a trigger factor tag in *Escherichia coli*. PceA was purified and denatured in aerobic condition. Subsequently, this protein was refolded in the presence of FeCl_3_, Na_2_S and cobalamin in anaerobic condition. The reconstituted PceA exhibited dechlorination ability for tetrachloroethene. UV-Vis spectroscopy has shown that it contains cobalamin and Fe-S clusters. Since this method requires anaerobic manipulation only in the reconstituting process and has a relatively high yield, it will enable further biochemical studies of RDases.

## Introduction

Organohalides are recalcitrant pollutants that have caused contamination of soils and groundwater in many sites around the world. Various bacteria derive their metabolic energy from dehalorespiration, which uses organohalides as terminal electron acceptors in anaerobic respiration (Holliger et al., 1998). Organohalide-respiring bacteria include *Dehalococcoides, Dehalogenimonas, Dehalobacter, Desulfitobacterium, and Sulfurospirillum*, as well as the organohalide-respiring members of the Deltaproteobacteria. Organohalide-respiring bacteria play critical roles in anaerobic bioremediation of sites contaminated by organohalides (Jugder et al., 2016). Organohalide-respiring bacteria are equipped with the conserved membrane-associated proteins, reductive dehalogenases (RDases), which catalyze reductive dehalogenation reactions resulting in the generation of lesser-halogenated compounds (Jugder et al., 2015). RDase is composed of two components, RdhA and RdhB. The catalytically active subunit RdhA contains one cobalamin and two Fe–S clusters (either two 4Fe–4S clusters or one 4Fe–4S and one 3Fe–4S cluster). There is one twin-arginine (TAT) signal sequence (RRXFXK) at the N-terminus, which mediates the transport of pre-folded RdhA into the periplasm. RdhA is associated with the outer side of the cytoplasmic membrane via the integral membrane protein RdhB.

Recent genome analyses of organohalide-respiring bacteria showed the existence of various RDase genes. Among these genes, there exist many RDase genes in genomes of *Dehalococcoides* spp. For example, 38 RdhA genes were identified in the genome of *D. mccartyi* strain MB (Low et al., 2015). However, the current understanding of RDase is limited to the results of studies on only several RDases, PceA (Schmitz et al., 2007; Sjuts et al., 2012), CprA (van de Pas et al., 1999, 2001), TceA (Magnuson et al., 2000), and vinyl chloride RDase (VcrA) (Muller et al., 2004; Parthasarathy et al., 2015). Existence of various RDases in *Dehalococcoides* spp. hampered the characterization of each RDase. It is difficult to culture organohalide-respiring bacteria for purification of RDases. Moreover, the heterologous expression of RDases is generally difficult because most RDases are oxygen-labile membrane-associated proteins. The heterologous expression of a few RDases was reported. Catabolic RDase BhbA from *Comamonas* is not oxygen sensitive and could be functionally expressed (Chen et al., 2013). PceA of *D. hafniense* strainY51 was overexpressed in a cobamide-producing bacterium *Shimwellia blattae* (Mac Nelly et al., 2014) and RdhA from *Nitratireductor pacificus* pht-3B (NpRdhA) in *Bacillus megaterium* (Payne et al., 2015). Recently, it was reported that heterologously expressed *Dehalococcoides* VcrA in *Escherichia coli*, was purified and reconstituted to its active form by the addition of hydroxocobalamin/adenosylcobalamin, Fe^3+^, and sulfide in the presence of mercaptoethanol (Parthasarathy et al., 2015). VcrA was obtained as a fusion protein with a maltose binding protein as an insoluble protein. In this method, all purification and reconstitution processes were performed in anaerobic conditions. However, the method was laborious, and the yield was insufficient.

In the present study, we tried functional expression and characterization of PceA from *Geobacter* sp. We expressed PceA with a fusion protein with the trigger factor tag and also the Strep-tag (TF-PceA-Strep). After purification and denaturation in 8 M urea in aerobic conditions, PceA was reconstituted with Fe and cobalamin in anaerobic condition. Thus, the reconstituted TF-PceA-Strep exhibited RDase activity on PCE.

## Materials and Methods

### Cloning and Expression of *PceA*

The *pceA* gene was obtained by PCR from the metagenome DNA of PCE dechlorinating bacterial consortium using the primers (5′-ATG GAT CGT AGA GAT TTT TTT AAA AAG GCA GC-3′ and 5′-CTA TGC CTT GTT CCA GAA GTC CG-3′). The amplified DNA was cloned into the T-vector pMD20 (TaKaRa Bio, Inc., Shiga, Japan) and subjected to DNA sequencing.

The full-length *pceA* was amplified using the primers (5′-CCC AAG CTT ATG GAT CGT AGA GAT TTT TTT AAA AAG G-3′ and 5′-CCC TCG AGC TAT GCC TTG TTC C-3′) and cloned into the HindIII/XhoI site of pET23b (Merck Millipore, Co., Billerica, MA, United States) (pET23b_PceA).

To express PceA as a fusion protein with TF, the gene was amplified with the primers (5′-CGA GCT CAT GGA TCG TAG AGA TTT TTT TAA AAA GG-3′ and 5′-CCC TCG AGC TAT GCC TTG TTC C-3′), and subcloned into the SacI/XhoI site of pCold TF vector (TaKaRa Bio, Inc.) (pCold TF-PceA). Then, the Strep-tag sequence was inserted at the C-terminus by the QuikChange method using the primers (5′-CGG ACT TCT GGA ACA AGG CAT GGA GCC ATC CGC AGT TTG AAA AGT AAG TCG ACC TGC AGT CTA GAT AG-3′ and 5′-CTA TCT AGA CTG CAG GTC GAC TTA CTT TTC AAA CTG CGG ATG GCT CCA TGC CTT GTT CCA GAA GTC CG-3′) (pCold TF-PceA-Strep).

Thus, the prepared plasmids (pET23b_PceA, pCold TF-PceA, and pCold TF-PceA-Strep) were used for the transformation of *E. coli* BL21 star (DE3). The transformed cells were grown in Luria-Bertani medium containing 100 μg/ml ampicillin at the specified temperatures. The cells were harvested by centrifugation at 5000 rpm for 15 min at 4°C.

### Purification of TF-PceA-Strep and Cofactor Reconstitution

Approximately 3 g wet weight of TF-PceA-Strep was re-suspended in 30 ml of suspension buffer (50 mM potassium phosphate pH 8.0 containing 0.5 M NaCl, 20% glycerol, 1mM PMSF). After cell disruption using an ultrasonic disruptor (UD-201, TOMY, Co., Tokyo, Japan), the suspension was centrifuged for 15 min at 14,000 rpm. The supernatant was discarded, and the pellet was resuspended in 15 ml of the denaturation buffer (suspension buffer + 8 M urea). Following another round of sonication, the suspension was stirred on ice for 1 h to solubilize the pellet. Centrifugation for 15 min at 4°C at 14,000 rpm was performed to remove remaining insoluble material.

The supernatant containing denatured TF-PceA-Strep was loaded onto the Ni-affinity column (His-Trap FF, GE Healthcare, Buckinghamshire, United Kingdom), which had been washed with five column volumes of the denaturation buffer and eluted with four column volumes of 250 mM imidazole in the denaturation buffer.

Next, the reconstitution of TF-PceA-Strep was performed under anaerobic conditions. All buffers were purged with 99.99% Ar and placed in the anoxic glove box before use. Denatured TF-PceA-Strep was mixed with the reduction buffer (100 mM Tris-HCl pH 7.5, 200 mM DTT) to 5 mM of DTT, and incubated for 30 min with stirring. Then, Fe buffer (100 mM Tris-HCl pH 7.5, 100 mM FeCl_3_) and S buffer (100 mM Tris-HCl pH 7.5, 30 mM Na_2_S) were added up to 50 mol excess of Fe and S to TF-PceA-Strep. After 90 min incubation with stirring, cyanocobalamin was added to a final concentration of 10 mg/ml. Next, TF-PceA-Strep solution was applied to a PD-10 column (GE Healthcare) equilibrated with the refolding buffer (50 mM Tris-HCl pH 8.0, 0.5 M NaCl, 20% glycerol, 0.2% CHAPS, 1 mM PMSF, and 10 mM DTT). The fractions containing reconstituted TF-PceA-Strep were collected.

### PceA Dechlorination Activity Assay

Enzyme activity experiments were performed in the anoxic sealed serum vials of 10 ml volume with butyl rubber stopper. In an anoxic vial, 3 ml of 50 mM Tris-HCl pH 8.5 containing 0.2 mg/ml of the reconstituted TF-PceA-Strep, 1 mM Ti(III) citrate as the electron donor and 0.4 mM methyl viologen as the electron mediator was added. The reaction was started by injecting PCE to approximately 100 μM PCE. Headspace samples (100 μl) were injected into the gas chromatograph (Shimadzu GC 2014 gas chromatograph, Shimadzu, Co., Kyoto, Japan) equipped with a DB-624 column (60 m length, 0.32 mm diameter, and 1.80 μm film thickness, Agilent Technology, Santa Clara, CA, United States) and a flame ionization detector.

### Mass Spectrometry

The band for TF-PceA-Strep in SDS-PAGE was excised and applied for the treatment by In-Gel Tryptic Digestion Kit (ThermoFisher Scientific, Co., Waltham, MA, United States). LC–MS/MS analysis of the digested peptides was performed using reversed-phase LC interfaced with a Q-TOF mass spectrometer (Bruker Daltonics, Co., Billerica, MA, United States). The digest peptides were separated using a PEGASIL ODS SP300-3 column (φ1 mm × 100 mm, 3 μm; Senshu Scientific, Co., Tokyo, Japan) eluted with a linear gradient of 0–100% buffer B (100% acetonitrile and 0.1% trifluoroacetic acid) in buffer A (0.1% trifluoroacetic acid in water) at a flow rate of 0.04 ml/min. MS and MS/MS data were acquired using the data-dependent top five method. The resulting MS/MS data were searched against the sequences of TF-PceA-Strep, using BioTools (Bruker Daltonics, Co.).

### UV-Vis Spectroscopy

UV-Vis absorbance spectra were recorded with a Varian CARY 50 UV-VIS Spectrophotometer (Agilent Technologies) in aerobic condition.

### Metal Analysis

Co and Fe content was analyzed by graphite furnace atomic absorption spectrometry (GFAAS). We used the Polarized Zeeman Atomic Absorption Spectrophotometer ZA3000 (Hitachi High-Technologies, Co., Tokyo, Japan) for GFAAS. At first, we made the calibration curves using the standard solutions of Fe and Co at the concentration of 0, 2.5, 5.0, 10, or 15 ppb in the Refolding Buffer (50 mM Tris-HCl pH 8.0, 0.5 M NaCl, 20% glycerol, and 0.2% CHAPS). Next, the reconstituted TF-PceA-Strep was diluted with 2% nitric acid. The diluted samples were applied for GFAAS analysis. The average values of triplicate measurements were used.

### Circular Dichroism (CD) Measurement

The secondary structure of the refolded TF-PceA was analyzed by CD. Protein samples were prepared in 2 mM Tris-HCl, pH 8.0 at 8.9 μM and then filtrated with cellulose acetate filter (0.2 μm). CD spectrum with a wavelength range of 205–260 nm was measured at 20°C by CD spectrometer (J-820, JASCO, Tokyo, Japan) using a 2 mm optical path length cuvette.

## Results

**FIGURE 1:**
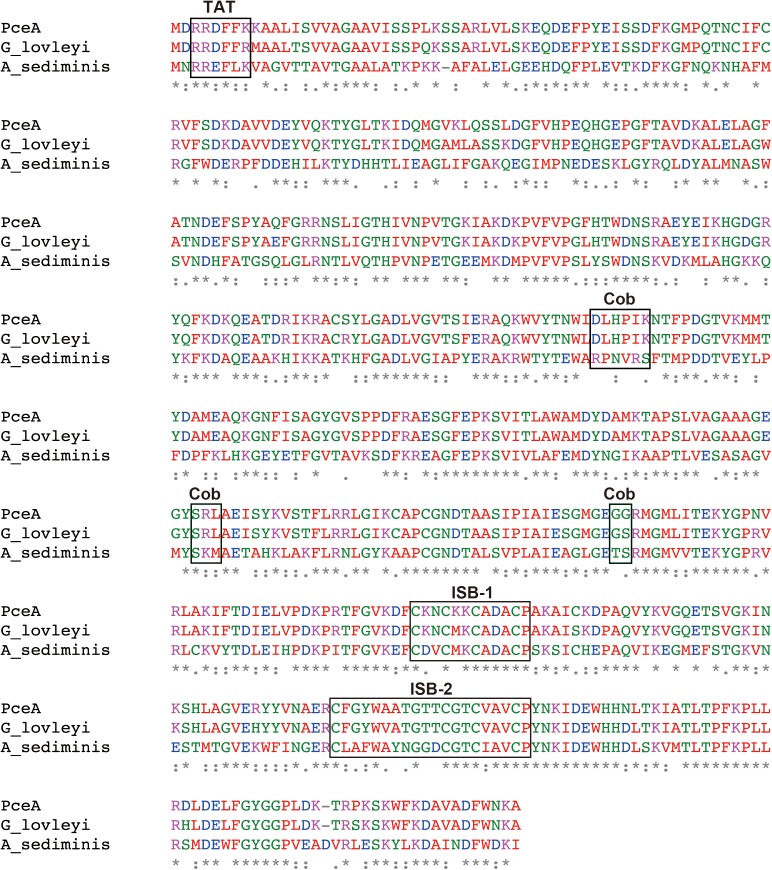
Amino acid sequence alignment for the PceA of this study and other homologous PceAs. Sequence alignment was performed by Clustal Omega. The identical amino acids are marked by asterisk. Double dot and dot designate similar amino acid residues. PceA (this study), G_lovleyi SZ (PceA from *Geobacter lovleyi* SZ, AFN20668.1), and A_sediminis (PceA from *Anaeromicrobium sediminis*, WP_095136311.1). TAT, Twin-arginine motif; ISB-1 and ISB-2, iron–sulfur cluster binding motif; Cob, Cobalamin binding motif.

Previously, we had constructed a PCE dechlorinating bacterial consortium. The consortium could dechlorinate tetrachloroethene (PCE) to *cis*-1,2-dichloroethene (*cis*-DCE) (**Supplementary Figure S1**). The 16S rRNA gene analysis of the metagenome showed the presence of *Dehalobacter* sp., *Geobacter* sp., *Sulfurospirillum* sp., *Clostridium* sp. and *Bacteroides* sp. Among these species, we speculated that *Dehalobacter* sp. or *Geobacter* sp. is responsible for dechlorination of PCE. Next, we attempted to amplify *pceA* genes from the metagenome using various primers designed from *pceA* genes of *Dehalobacter* sp. and *Geobacter* sp. We could obtain the full-length *pceA* gene, which exhibits significantly high sequence identity with that of *G. lovleyi* SZ (**Figure 1**). The Nucleotide sequence is available in the DDBJ/EMBL/GenBank databases under the accession number, LC342077. *G. lovleyi* SZ was obtained from the non-contaminated creek sediment microcosms based on their ability to derive energy from acetate oxidation coupled to PCE-to-*cis-*DCE dechlorination (Sung et al., 2006). The putative RDase from *Anaeromicrobium sediminis* also showed significant sequence identity. The sequence alignment of the three RDases is shown in **Figure 1**. In the N-terminal region, there exists the consensus motif of twin-arginine signal sequence, RRXFXK. Two iron–sulfur cluster binding (ISB) sites are also conserved. The first ISB, CKNCKKCADACP, corresponds to the conserved consensus sequence CXXCXXCXXXCP, found in bacterial ferredoxins (Bruschi and Guerlesquin, 1988). The second ISB is partly different from the consensus one. The latter motif, CXXCXXXCP, is conserved, but the first Cys residue is separated from the second Cys residue by 10 amino acid residues. The consensus sequence for cobalamin binding, DXHXXG...SXL...GG is partially conserved in PceA (Ludwig and Matthews, 1997). However, there are sequence variations in the corresponding sequences in the homologous putative PceAs.

**FIGURE 2:**
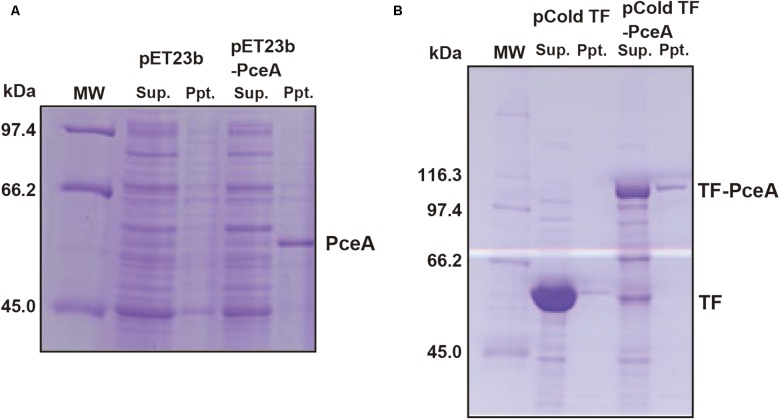
Expression of PceA and TF-PceA in *Escherichia coli*. The supernatant and precipitate of the crude extracts of *E. coli* cells harboring designated plasmids are analyzed by SDS-PAGE. **(A)** pET23b and pET23b-PceA **(B)** pCold TF and pCold TF-PceA.

To express the *Geobacter* PceA in *E. coli*, the *Geobacter pceA* was cloned into the pET23b vector (pET23b-pceA) and used to transform *E. coli* BL21 star (DE3). PceA was obtained as an aggregate in the insoluble fraction (**Figure 2A**). Next, we tried to express PceA as a fusion protein with the trigger factor tag (TF-PceA). TF-PceA was obtained as a soluble protein at the induction of protein expression at 18°C (**Figure 2B**). *E. coli* cells expressing TF-PceA were collected and disrupted by the lysozyme treatment at the anaerobic conditions in a glove box. The total cell lysate was applied for dechlorination assay for PCE. Unexpectedly, no PCE dechlorination activity was observed. We further tried to purify TF-PceA in aerobic conditions using the histidine tag attached to TF tag. However, TF-PceA was subjected to protease digestion during purification by Ni affinity chromatography. Next, we added Strep-tag at the C-terminus (TF-PceA-Strep). TF-PceA-Strep was purified by affinity chromatography using Strep-tactin column and size exclusion chromatography. In this case, no protease digestion was observed. In the mass spectroscopic analysis, the proteolytic peptide fragments covered approximately 40% of TF-PceA-Strep (**Supplementary Figure S2**). Since UV-Vis spectrum of the purified TF-PceA-Strep lacked the peak for cobalamin, we concluded that the active center was not correctly formed in TF-PceA-Strep.

We speculated that although active PceA was obtained in *E. coli*, it is difficult to purify, because all procedures must be performed in anaerobic conditions. Thus, we decided to reconstitute active PceA *in vitro*. Since it was difficult to reconstitute PceA *in vitro*, we used TF-PceA-Strep. We expressed TF-PceA-Strep as an insoluble protein by culturing recombinant *E. coli* at 37°C. Then, the precipitated proteins were solubilized by buffer containing 8 M Urea. TF-PceA-Strep was purified by nickel affinity chromatography in the presence of 8 M urea. The denatured TF-PceA-Strep was applied for reconstitution in the anaerobic glove box. Denatured TF-PceA-Strep was mixed with DTT, FeCl_3_, Na_2_S and cobalamin in denaturation buffer, and the mixture was loaded onto a PD-10 desalting column equilibrated with the refolding buffer. TF-PceA-Strep was expected to reconstitute by removing urea, and unincorporated excess cofactors were also removed. CD spectrum shows that the reconstituted TF-PceA-Strep takes the folded conformation (**Supplementary Figure S3**).

**FIGURE 3:**
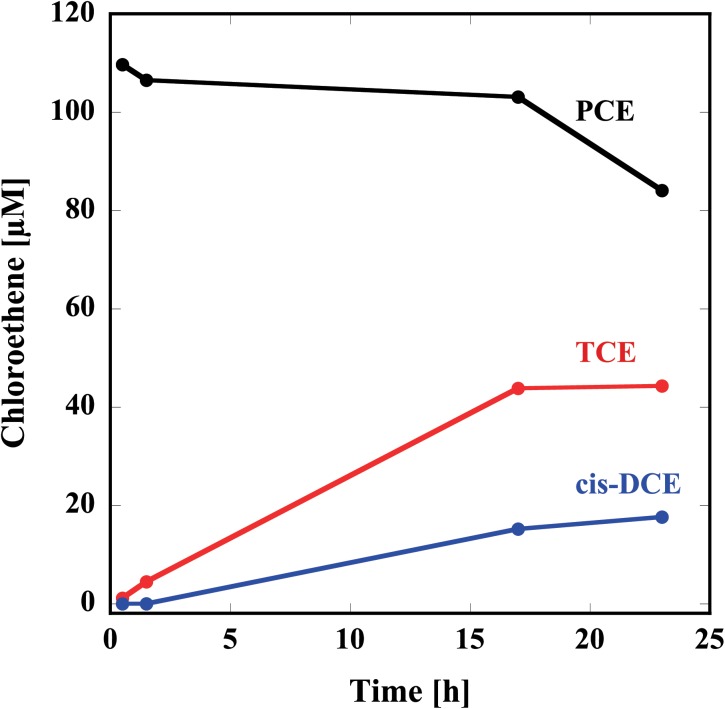
Reduction of PCE to TCE and *cis-*DCE by the reconstituted TF-PceA-Strep. Time-course concentration changes of PCE (black), TCE (red), and *cis-*DCE (blue) are plotted.

Thus, prepared reconstituted TF-PceA-Strep was used for the dechlorination assay for PCE. The reconstituted TF-PceA-Strep was mixed with PCE in the presence of Ti(III) citrate as the electron donor and methyl viologen as the electron mediator. As shown in **Figure 3**, in the presence of TF-PceA-Strep, PCE decreased with the increase of TCE and *cis-*DCE. However, a decrease in PCE and the appearance of TCE and *cis-*DCE were not observed in the control experiments using a protein unrelated to reductive dehalogenation, Hsp104 from a thermophilic fungus.

**FIGURE 4:**
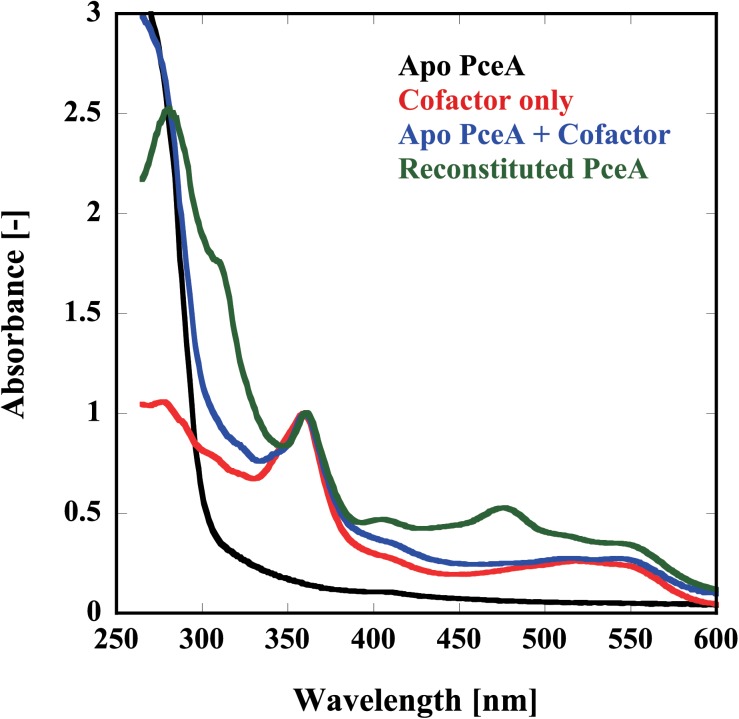
Reconstitution of the catalytic center in TF-PceA-Strep observed by UV-Vis spectrum. UV-Vis spectra of apo-TF-PceA-Strep (refolded without cofactor) (black), cofactors only (red), apo-TF-PceA-Strep + cofactor (blue), and the reconstituted TF-PceA-Strep (green) are shown. The spectra were normalized by the absorbance of 360 nm.

Finally, we examined the reconstitution of the catalytic centers by UV-Vis spectrophotometry and metal analysis. Because of the apparatus limitation, we performed UV-Vis spectrophotometry in aerobic conditions. Before reconstitution, no peaks derived from the cofactor were detected. Alternatively, peaks attributed to each cofactor were detected from the sample after reconstruction; [4Fe–4S] at 420 nm, Co(I) at 360 and 550 nm, and Co(II) at 310 nm (**Figure 4**). To obtain the quantitative data, the metal content of the reconstituted TF-PceA-Strep was analyzed by GFAAS (**Table 1**). The molar ratio of TF-PceA-Strep:Co:Fe was 1:1.4:10. This ratio was close to the theoretical value of 1:1:8, suggesting that TF-PceA-Strep and cofactor coexist with values close to the theoretical values.

**Table 1:** Fe and Co content in the reconstituted TF-PceA-Strep.

	TF-PceA-Strep	Co	Fe
Quantity (nmol)	2.645	3.77	28.2
Ratio	-	1.42	10.7


## Discussion

There are many genes encoding RDases in the genome of organohalide-respiring bacteria, including *Dehalococcoides* spp. However, most of them have not been functionally characterized. Existence of multi RDases in a bacterium makes it also difficult to examine the substrate specificities of them. Since most RDases are membrane proteins labile for aerobic condition, it is generally difficult to obtain recombinant ones. The catalytic subunit, RdhA, is not embedded in the membrane. Therefore, it is possible to obtain RdhA as a soluble protein. However, generally, RdhA does not fold correctly in *E. coli* probably due to its partially hydrophobic nature to interact with the membrane bound RdhB.

PceA from *D. restrictus* was expressed as a fusion protein with the trigger factor (TF) tag, and purified under anaerobic conditions (Sjuts et al., 2012). Recently, heterologous production of RdhA of VcrA from *D. mccartyi* strain VS was reported (Parthasarathy et al., 2015). In both cases, UV-Vis and electron paramagnetic resonance spectroscopy revealed that catalytic centers are correctly constituted. In their methods, all purification procedures were conducted in anaerobic conditions. Therefore, it is difficult to perform in laboratories with ordinary instruments. Moreover, the yield is relatively small for detailed studies. Our idea was to obtain denatured RdhA in aerobic conditions and refold it with their cofactors.

In this study, we tried to express and characterize the PceA. The *pceA* gene is significantly homologous to that of *G. lovleyi*. This gene also showed high homology to the putative RDase from *A. sediminis*. The sequence identity was estimated to be 51%. The sequence identity with the other putative PceA is relatively modest. The identity with the PceA from *Dehalobacter* sp. E1 (EQB20894.1) is approximately 37%. The two ISB motifs are conserved with that of PceA from *Dehalobacter* sp. E1. However, cobalamin binding motif is not well-conserved. Because *Geobacter* sp. and *Dehalobacter* sp. seemed to be responsible for dechlorination of PCE in the original bacterial consortium, we concluded that the gene was originated from a *Geobacter* sp.

We expressed PceA as a fusion protein with the trigger factor tag and also the Strep-tag (TF-PceA-Strep). TF-PceA-Strep was denatured by buffer containing 8 M urea and purified by affinity chromatography using the histidine tag in trigger factor tag. Although TF-PceA-Strep was only partially purified in this study, it is possible to perform further purification using different types of chromatography. The reconstitution of TF-PceA-Strep was performed by size exclusion chromatography using a buffer containing cofactors in anaerobic conditions. Since TF-PceA-Strep can refold easily, this process requires only a small column. The reconstituted TF-PceA-Strep could convert PCE to *cis-*DCE via TCE. UV-Vis and GFAAS suggested binding of TF-PceA-Strep and a cofactor. The measured metal content of the reconstituted sample was partially larger than the theoretical value. This result is probably observed because the separation of free Fe and cobalamin by PD-10 is not sufficient.

We have established a heterologous platform to produce the TF-PceA-Strep enzyme, which can be reconstituted in its active form. It was expected that the function and structure of RDase would be clarified by the crystallization of RDase under anaerobic conditions. Further study is needed to understand all of the components related to the synthesis and activity of RdhA, with continued future research efforts investigating the accessory genes and their products, such as transcriptional regulators (PceC, MarR), anchoring proteins (RdhB or PceB), and maturation proteins (e.g., PceT). This platform may be employed for obtaining recombinant proteins with the same characteristics as RDase.

## Author Contributions

RNA performed all data collections. TOB, YH, TOG, and KN assisted the data collection. RNO constructed PCE dechlorinating bacterial consortium and assisted data collection. MY designed the study and wrote the manuscript.

## Conflict of Interest Statement

The authors declare that the research was conducted in the absence of any commercial or financial relationships that could be construed as a potential conflict of interest.
