# Supplementary material for: Functional Expression and Characterization of Tetrachloroethene Dehalogenase From Geobacter sp
Source: Front Microbiol. 2018 Aug 10;9:1774. doi: 10.3389/fmicb.2018.01774 (PMC6095959; doi:10.3389/fmicb.2018.01774)
Supplement: Supplementary file 1 [file Data_Sheet_1.PDF]

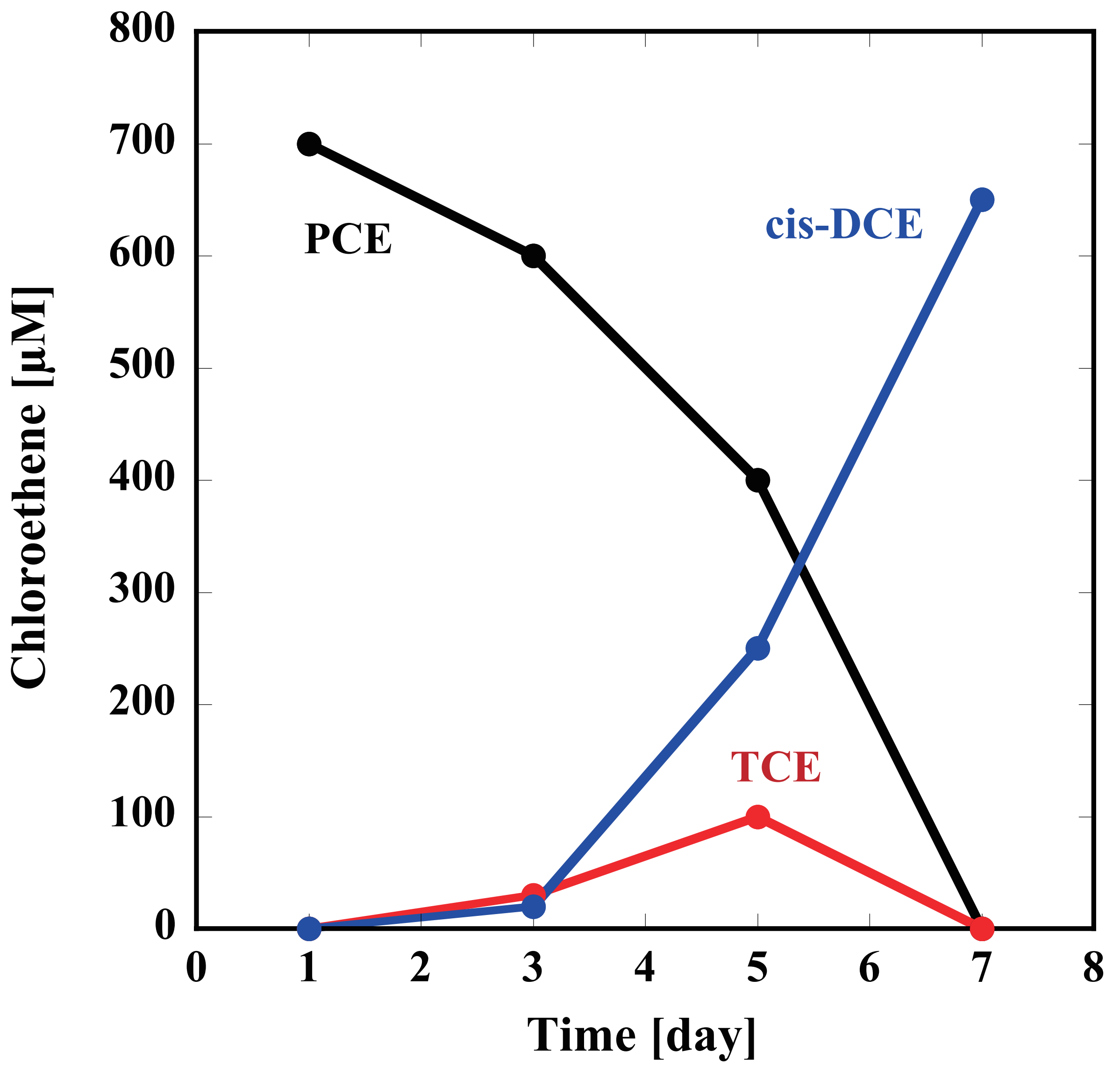

Supplementary Fig.S1

Reductive dechlorination of PCE by the consortium.

Supplementary Fig. S2

The peptide sequences of TF-PceA-Strep identified by Mass spectrometry

MNHKVHHHHHHMQVSVETTQGLGRRVTITIAADSIETAVKSELVNVAKKVRIDGFRKGKV
PMNIVAQRYGASVRQDVLGDLMSRNFIDAIIKEKINPAGAPTYVPGEYKLGEDFTYSVEF
EVYPEVELQGLEAIEVEKPIVEVTDADVDGMLDTLRKQQATWKEKDGAVEAEDRVTIDFT
GSVDGEEFEGGKASDFVLAMGQGRMIPGFEDGIKGHKAGEEFTIDVTFPEEYHAENLKGK
AAKFAINLKKVEERELPELTAEFIKRFGVEDGSVEGLRAEVRKNMERELKSAIRNRVKSQ
AIEGLVKANDIDVPAALIDSEIDVLRRQAAQRFGGNEKQALELPRELFEEQAKRRVVVGL
LLGEVIRTNELKADEERVKGLIEEMASAYEDPKEVIEFYSKNKELMDNMRNVALEEQAVE
AVLAKAKVTEKETTFNELMNQQASAGLEVLFQGPSAGLVPRGSGGIEGRHMELMDRRDFF
KKAALISVVAGAAVISSPLKSSARLVLSKEQDEFPYEISSDFKGMPQTNCIFCRVFSDKD
AVVDEYVQKTYGLTKIDQMGVKLQSSLDGFVHPEQHGEPGFTAVDKALELAGFATNDEFS
PYAQFGRRNSLIGTHIVNPVTGKIAKDKPVFVPGFHTWDNSRAEYEIKHGDGRYQFKDKQ
EATDRIKRACSYLGADLVGVTSIERAQKWVYTNWIDLHPIKNTFPDGTVKMMTYDAMEAQ
KGNFISAGYGVSPPDFRAESGFEPKSVITLAWAMDYDAMKTAPSLVAGAAAGEGYSRLAE
ISYKVSTFLRRLGIKCAPCGNDTAASIPIAIESGMGEGGRMGMLITEKYGPNVRLAKIFT
DIELVPDKPRTFGVKDFCKNCKKCADACPAKAICKDPAQVYKVGQETSVGKINKSHLAGV
ERYYVNAERCFGYWAATGTTCGTCVAVCPYNKIDEWHHNLTKIATLTPFKPLLRDLDELF
GYGGPLDKTRPKSKWFKDAVADFWNKAWSHPQFEK*

The peptide sequences identified by Mass spectrometry are marked by red font.

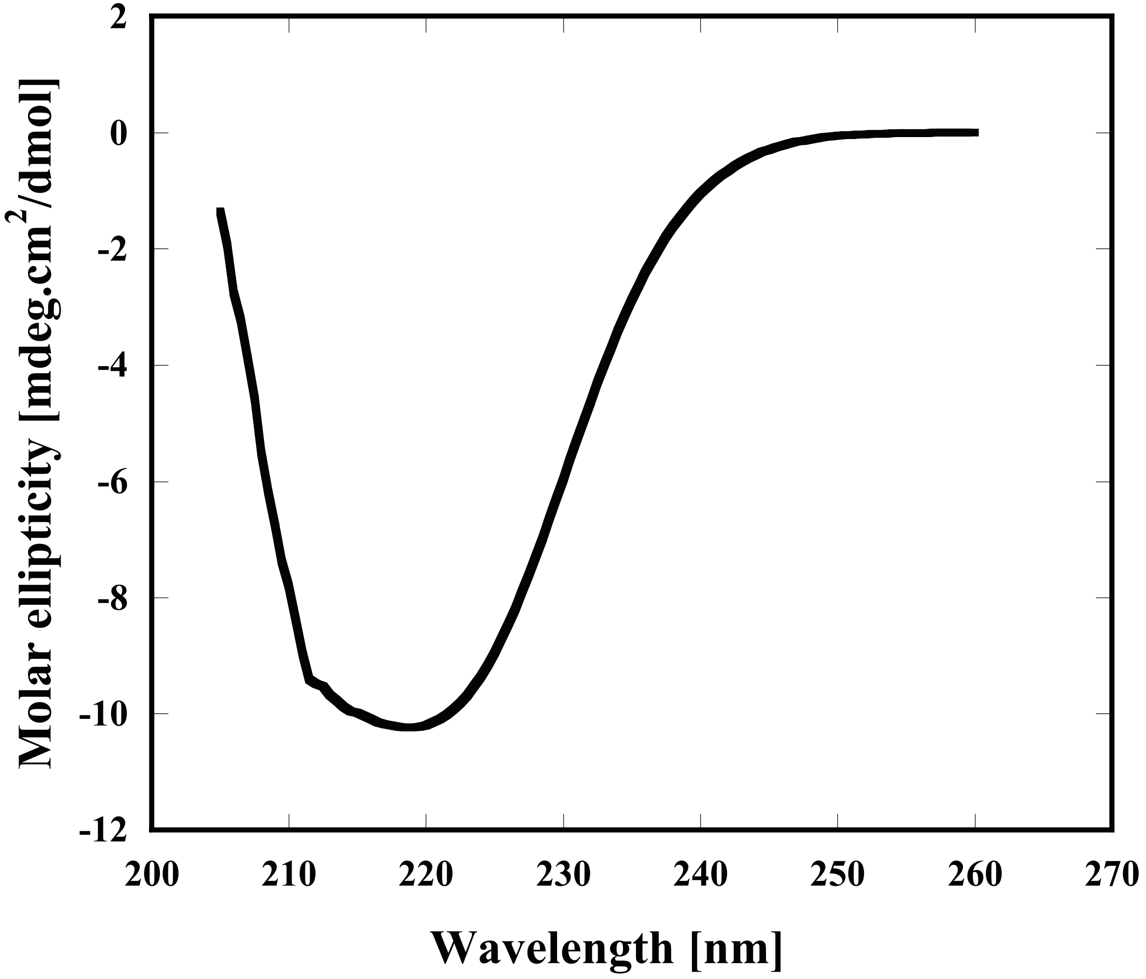

Supplementary Fig. S3

CD spectrum of the refolded TF-PceA
